# *Histoplasma* yeast and mycelial transcriptomes reveal pathogenic-phase and lineage-specific gene expression profiles

**DOI:** 10.1186/1471-2164-14-695

**Published:** 2013-10-10

**Authors:** Jessica A Edwards, Chenxi Chen, Megan M Kemski, Jinnan Hu, Thomas K Mitchell, Chad A Rappleye

**Affiliations:** 1 The Department of Microbiology, Ohio State University, 484 W. 12th Ave., Columbus, OH 43210, USA; 2 The Department of Microbial Infection and Immunity, Ohio State University, 484 W. 12th Ave., Columbus, OH 43210, USA; 3 The Department of Plant Pathology, Ohio State University, 484 W. 12th Ave., Columbus, OH 43210, USA

**Keywords:** *Histoplasma*, Transcriptome, Dimorphism, Fungal pathogenesis

## Abstract

**Background:**

The dimorphic fungus *Histoplasma capsulatum* causes respiratory and systemic disease in mammalian hosts by expression of factors that enable survival within phagocytic cells of the immune system. *Histoplasma*’s dimorphism is distinguished by growth either as avirulent mycelia or as pathogenic yeast. Geographically distinct strains of *Histoplasma* differ in their relative virulence in mammalian hosts and in production of and requirement for specific virulence factors. The close similarity in the genome sequences of these diverse strains suggests that phenotypic variations result from differences in gene expression rather than gene content. To provide insight into how the transcriptional program translates into morphological variation and the pathogenic lifestyle, we compared the transcriptional profile of the pathogenic yeast phase and the non-pathogenic mycelial phase of two clinical isolates of *Histoplasma*.

**Results:**

To overcome inaccuracies in ab initio genome annotation of the *Histoplasma* genome, we used RNA-seq methodology to generate gene structure models based on experimental evidence. Quantitative analyses of the sequencing reads revealed 6% to 9% of genes are differentially regulated between the two phases. RNA-seq-based mRNA quantitation was strongly correlated with gene expression levels determined by quantitative RT-PCR. Comparison of the yeast-phase transcriptomes between strains showed 7.6% of all genes have lineage-specific expression differences including genes contributing, or potentially related, to pathogenesis. GFP-transcriptional fusions and their introduction into both strain backgrounds revealed that the difference in transcriptional activity of individual genes reflects both variations in the cis- and trans-acting factors between *Histoplasma* strains.

**Conclusions:**

Comparison of the yeast and mycelial transcriptomes highlights genes encoding virulence factors as well as those involved in protein glycosylation, alternative metabolism, lipid remodeling, and cell wall glycanases that may contribute to *Histoplasma* pathogenesis. These studies lay an essential foundation for understanding how gene expression variations contribute to the strain- and phase-specific virulence differences of *Histoplasma.*

## Background

Pulmonary infections with the dimorphic fungal pathogen *Histoplasma capsulatum* constitute one of the most common respiratory mycoses, affecting both immunocompromised as well as immunocompetent individuals [1,2]. *Histoplasma* is acquired by inhalation of infectious conidia, which are produced by the environmental mycelial form of the fungus. The dimorphism of *Histoplasma* is evident within the mammalian host where the elevated temperature causes differentiation of the conidia into the virulent yeast form. The yeasts infect, survive, and replicate within lung alveolar macrophages. The extent of histoplasmosis disease results from the net contributions of initial inoculum size, the inherent virulence of the strain, and the immune status of the host [3].

The differentiation of *Histoplasma* into yeasts and expression of the yeast-phase transcriptional program are necessary for virulence. *Histoplasma* cells genetically or chemically prevented from transitioning into yeast are avirulent, highlighting the essentiality of differentiation to the pathogenic phase [4-7]. However, it is most likely the expression of yeast-phase-specific genes, rather than the morphology itself, that contributes to *Histoplasma* virulence. Accordingly, most *Histoplasma* virulence factors identified to date are restricted to pathogenic-phase yeast cells [8-12].

The *H. capsulatum* species is constituted of several geographically and phylogenetically distinct groups. Two clades, the North American clade 2 (NAm2; e.g., clinical isolate G217B) and the Panamanian clade (Pan; e.g., clinical isolate G186A) [13,14], typify the diversity among *Histoplasma* strains both at the genomic and phenotypic levels. Both strains are virulent, although in murine models of histoplasmosis, G217B infection results in higher organ fungal burdens and increased lethality compared to G186A [15-17]. The G217B genome is roughly 30% larger than the G186A genome (41.0 megabases vs. 30.4 megabases, respectively). Most of the excess DNA in G217B is located in intergenic, repetitive DNA. Both genomes are predicted by *in silico* analyses of the genome sequence to encode between 9,000 and 10,000 genes (http://www.genome.wustl.edu; http://www.broadinstitute.org). The only differences in gene content determined to date, are the high-affinity iron transport genes, *FET3* and *FTR1*, which are found in the G186A genome but not the G217B genome [18].

The close similarity in gene content and in coding sequences among *Histoplasma* strains suggests that phenotypic differences likely result from differences in gene expression rather than variations in gene content. Two examples of known virulence factors clearly illustrate this. First, most *Histoplasma* lineages, including G186A, have cell walls containing α-glucan and rely on production of this polysaccharide for disease establishment [8]. In the G186A strain, deletion of the α-glucan synthase gene *AGS1* causes attenuation due to exposure of immunostimulatory cell wall β-glucans that are normally masked by α-glucan [19]. In contrast to G186A, the cell walls of many North American isolates, represented by G217B, lack α-glucan. Although G186A and G217B have nearly identical α-glucan synthase (*AGS1*) coding sequences, production α-glucanis absent in G217B during yeast-phase growth. In G217B, the *AGS1* promoter is interrupted by a 2.7-kb insertion of repetitive DNA elements that alters expression levels of the synthase. Despite this, G217B remains virulent, suggesting that this strain uses an alternative mechanism to circumvent the need for α-glucan [15]. The second example of expression-based phenotypic differences between *Histoplasma* strains is the *YPS3* gene, which encodes a yeast phase specific factor related to the *Blastomyces* Bad1 protein [20-22]. The genomes of both G186A and G217B contain the *YPS3* gene, but only G217B yeasts produce the Yps3 protein, which contributes to G217B virulence [23,24]. Similar to α-glucan, differential production of Yps3 appears to result from transcriptional regulation since placing the *YPS3* gene under control of an ectopic promoter in the G186A background is sufficient to restore Yps3 protein production [21].

To better understand how gene expression differences between *Histoplasma* strains translate into phenotypic differences including yeast virulence, a more complete examination of gene expression profiles is needed. Past technologies for defining the identity and quantity of all transcripts expressed by an organism have included both hybridization-based (e.g., microarrays) and sequence-based (e.g., Sanger sequencing of cDNA or EST libraries) approaches [25]. Hybridization-based approaches have been used to study differences in *Histoplasma* expression between non-pathogenic (mycelial) phase and pathogenic (yeast) phases [26,27] as well as during nitrosative stress [28]. Both of these studies were limited to intra-strain expression differences. Recently, a microarray-based analysis of two strains documenting mycelial, yeast, and conidial gene expression was determined [29]. The analytical power of microarrays, however, is limited since they are highly dependent on the accuracy of the predicted gene sets. Inaccurate gene structures derived from ab initio predictions misses or mispredicts genes resulting in errors in the annotations and subsequent microarray data. In contrast, next-generation sequencing-based transcriptome determination defines genes directly from experimentally derived mRNA sequence evidence. Furthermore, there is no upper limit to the expression level with the number of mapped reads being highly correlated to actual gene expression level [25].

In this study, we use next-generation sequencing (i.e., RNA-seq) of *Histoplasma* G186A and G217B yeast and mycelial mRNAs to profile the respective pathogenic and non-pathogenic-phase transcriptomes and to identify interstrain pathogenic-phase expression differences that may contribute to variations in virulence. Furthermore, we show for a subset of differentially expressed genes, that differences in expression result from both cis- and trans-acting factors that affect promoter activity in the different genetic backgrounds. These findings will improve our understanding of the mechanisms underpinning morphological, biochemical, and virulence differences among strains of *Histoplasma capsulatum*.

## Results

### Determination of the G186A and G217B *Histoplasma* transcriptomes using RNA-seq

To provide an experimental-evidence-based annotation of the *Histoplasma* genome, we used RNA-seq methodology to construct gene models with transcriptional support. For a more comprehensive gene definition, we sequenced the mRNAs from pathogenic-phase (yeast) and non-pathogenic-phase (mycelia) *Histoplasma* cells, the two distinguishing lifestyles of this dimorphic fungal pathogen. In addition, RNAs from two strains of *Histoplasma* (G186A and G217B), representing the Pan and NAm2 clades, respectively, were analyzed. These two strains are clinical isolates of *Histoplasma*, have complete genome sequences (http://www.genome.wustl.edu; http://www.broadinstitute.org), and are the two strains for which molecular genetic methodologies have been established [8,9,15,30-34]. For yeast-phase RNA samples, strains were grown to late-exponential phase (approximately 72 hours) in conditions approximating the mammalian host environment (growth at 37°C in 5% CO_2_ / 95% air). The growth of two cultures for biological replicate RNA samples was nearly identical as measured by yeast culture turbidity (data not shown). For mycelia RNA samples, strains were grown in liquid medium at 25°C in normal air without shaking until sufficient mycelial biomass formed (approximately 3 weeks). The RNA integrity number (RIN) [35] for all RNA samples used was ≥ 8.5. Next generation sequencing (Illumina RNA-seq) of the eight mRNA libraries (two biological replicates for each condition) yielded approximately 118 million paired-end reads for derivation of the *Histoplasma* set of expressed genes (38.6 million and 79.5 million reads for yeasts and mycelia, respectively).

### Experimental-evidence-based annotation of the *Histoplasma* genome

**Figure 1:**
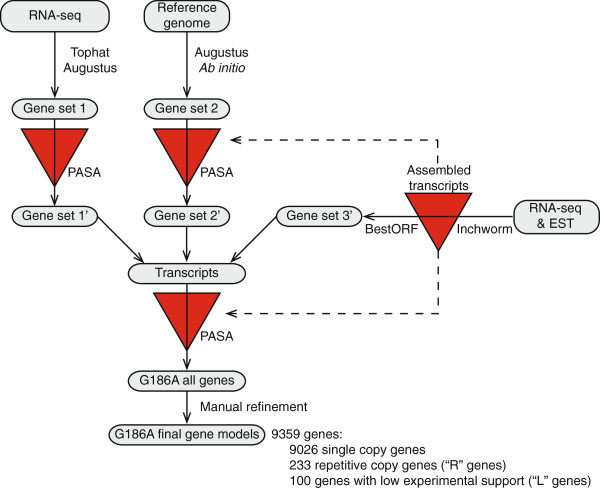
**Transcriptional evidence-based pipeline for assembly of *Histoplasma *G186A gene models.** RNA-seq data was used to generate spliced alignment models to the reference genome (gene set 1 and 2) as well as de novo assembly into contiguous transcripts (gene set 3′). Gene sets 1 and 2 were updated by PASA (red triangles) using the de novo transcript assembly. The three gene sets were integrated and subjected to an additional update using the de novo mRNA assembly. The resultant gene set was manually refined after inspection of the models for genes with unusual characteristics (introns greater than 350 bp or intergenic distances less than 500 bp). The final gene models were subsequently categorized as single copy genes, repetitive genes, or genes with low experimental support (gene models based on low numbers of RNA-seq reads).

The transcriptome data was first used to determine gene structures for G186A as the G186A genome contains much less repetitive DNA than the G217B genome. Gene structures were mapped onto the G186A reference genome by a bioinformatics pipeline that incorporated reference-genome-based mRNA reads alignment and de novo transcript assembly (Figure 1). Yeast and mycelia mRNA reads were aligned to the G186A reference genome using the spliced alignment tool Tophat [36]. For yeast, 56.8% of reads (10.8 million) were of sufficient quality for processing by Tophat analysis, and 84.9% of these were aligned to the reference genome. For mycelia, 60.9% of reads (20.0 million) were processed with 78.8% aligning to the reference genome. The exact exon and intron boundaries were used to inform gene structure determination through the eukaryotic gene predictor Augustus [37,38]. Separately, the RNA-seq short reads were assembled into transcript contigs de novo (i.e., independent of the reference genome sequence) using Inchworm [39] and open reading frames extracted from the transcripts with BestORF (Molquest package, Softberry). The de novo transcript assembly was input into PASA [40] to refine the alignment-based gene models with mRNA evidence (Figure 1). The three data sets were integrated and the gene models were subjected to a second update with evidence from the de novo transcript assembly reads using PASA. Lastly, the gene structures were interrogated for intergenic distances smaller than 500 bp or for introns greater than 350 bp based on known *Histoplasma* gene and promoter characteristics. These unusual genes structures were manually refined as appropriate. The final set of gene structures was annotated for single-copy genes or for repetitive genes (genes with 2 or more BLAST matches to the reference genome with e-values < 10^-40^; designated with ’R” in the accession number). In addition, genes with low experimental support (due to very low mRNA coverage; FPKM values less than 0.1 in all four libraries, see below) were identified (designated with “L” in the accession number). Overall, 9359 gene structures were identified which included 9026 single-copy genes, 233 repetitive genes, and 100 genes with low mRNA support.

Due to the high similarity of the genomes of G186A and G217B and the increased complexity of the G217B genome from the large amount of repetitive DNA, we used G186A gene structures to inform construction of the G217B genes. Short reads in the four G217B mRNA libraries (2 yeast-phase and 2 mycelial-phase) were aligned to the G186A reference genome using Tophat with relaxed parameters to account for the nucleotide variation between strains. We allowed for 6 mismatches in the 75-bp G217B short reads, providing at least 92% sequence identity, which is similar to the identity of known orthologous genes between strains (identity ranges from 93% to 99%). Using these parameters, 72% of the processed reads from each G217B library were matched to the G186A reference genome with high confidence and these were used to derive the base G217B gene set. As some genes unique to the G217B genome or only expressed by G217B cells would be missed, the reference-based alignment and de novo transcript assembly pipeline (Figure 1) was then applied to the remaining high quality G217B reads that were not directly matched to the G186A transcriptome. This identified an additional 62 G217B genes, which were added to the G217B models to derive the final G217B gene set of 9004 genes.

**Figure 2:**
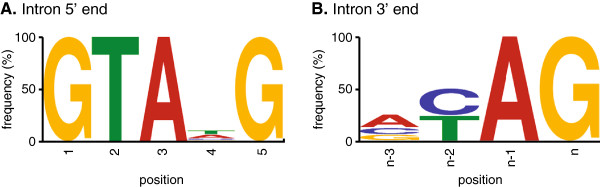
**Derived consensus of intron splicing signals.** The intron sequences common to the intron 5′ end **(A)** and the intron 3′ end **(B)** of *Histoplasma* genes. The vertical height of the nucleotide at each position indicates the relative nucleotide frequency (y-axis).

To identify general characteristics of *Histoplasma* gene loci, we queried the G186A total gene set for common features and motifs. The overall gene density in G186A is approximately 3 genes per 10 kb with an average gene length of 2041 bp, although there is a very broad range in exon and gene sizes (Table 1). 77% of genes have at least one intron. Introns are relatively short in *Histoplasma* with a median size of 82 and an average size of 103 base pairs. We used the defined gene structures in the yeast- and mycelial-phase transcriptomes to derive a consensus splicing signal. 15 base pairs at both ends of all introns were extracted and the most frequent 5′ and 3′ splicing signal motifs determined using the motif finder MEME [41]. The consensus splicing signals were GTA[A/T]G at the 5′ end of the intron (Figure 2A) and [C/T]AG at the 3′ end of the intron (Figure 2B), consistent with the intron 5′ GT and 3′ AG of eukaryotic splicing mechanisms [42].

**Table 1:** G186A gene statistics

	**Average**	**Middle 90% range**
**Gene length (bp)**	2041	333 - 4857
**Exons per gene**	3.0	1 - 7
**Exon length (bp)**	587	39-2103
**Introns per gene**	2.6	1 - 6
**Intron length (bp)**	104	54 - 237
**Gene density (per kb)**	3.1	N/A

### RNA-seq improved gene annotation for G186A

**Figure 3:**
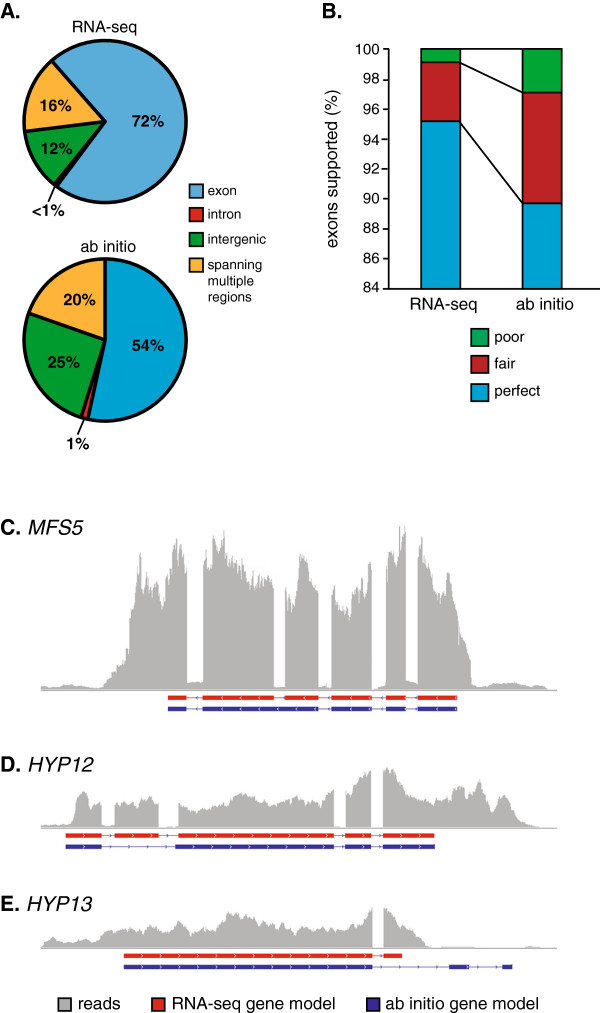
**Comparison of RNA-seq-derived gene models with *Histoplasma *ab initio gene predictions.** The accuracy of the RNA-seq-derived and ab initio gene models for G186A were measured as the frequency of mRNA reads that match the modeled gene structures **(A)**, the percentage of exon structures with mRNA experimental support **(B)**, and direct sequencing of mRNAs **(C**-**E)**. **(A)** Percentages indicate the number of cDNA library reads that match to exons (blue), introns (red), intergenic regions (green), or spanning multiple regions (yellow) in the RNA-seq-derived or ab initio gene set models. **(B)** Accuracy of the exon definition is indicated by the percentage of exons with perfect support (blue; at least 99% of the exon length is covered by mRNA reads), fair support (red; 70% to 99% of the exon length is covered by mRNA reads), or poor support (green; less than 70% of the exon length is covered by mRNA reads). **(C**-**E)** Schematics of gene structures are shown as exons (horizontal boxes below the x-axis) for RNA-seq-derived models (red) and the ab initio predictions (blue). The horizontal represents the genome sequence in that interval. Vertical histogram (grey bars) depicts the frequency of mRNA reads that match that particular region of the genome sequence. Models are depicted for the *MFS5* gene **(C)** that encodes an *MFS*-family transporter, the *HYP12* gene **(D)** and the *HYP13* gene **(E)**, two genes encoding factors of unknown function.

To determine the improvement in accuracy of the gene definitions resulting from RNA-seq, we compared our G186A gene models with the current ab initio G186A gene predictions (http://www.broadinstitute.org/annotation/genome/histoplasma-_capsulatum/MultiHome.html). Transcriptome sequencing yielded 126 more genes. The total length of exon regions from RNA-seq is 17.3 Mb (56.7% of the genome), compared with 13.8 Mb (45.2% of the genome) in the ab initio predictions. To further compare the sensitivity of the gene definitions from RNA-seq with the ab initio gene models, we analyzed where mRNA reads aligned in the respective gene models (RNA-seq based or ab initio predictions). A read with > 95% of its length aligning to a region defined as an exon was considered as strong experimental validation of the locus. By these strict criteria, 72% of the G186A mRNA reads matched the RNA-seq-derived gene structures (Figure 3A). In contrast, only 54% of the mRNA reads matched the ab initio gene predictions. A similar proportion of reads aligned to intron regions in both data sets (0.47% and 1% for RNA-seq and ab initio gene models, respectively). Reads aligning to intronic or overlapping multiple region classifications are not unexpected due to partially processed RNAs in the transcriptome library and the possibility of alternative splicing events [36]. This indicates the mRNA evidence more strongly supports the RNA-seq-derived gene set compared to the ab initio gene predictions. In addition, there are notable differences in the introns defined in the RNA-seq based gene structures and the ab initio predictions. The RNA-seq data shows 90% of introns are between 54 and 237 bp in size (Table 1). The ab initio predictions are slightly broader with the middle 90% ranging from 51 to 365 base pairs. Notably, the ab initio predicted gene set introns have an overall range from 11 to 1566 bp in size, which includes 1597 introns larger than 300 bp in size. These longer and shorter intron sizes in the ab initio predictions are not supported by the mRNA reads suggesting prediction errors in the ab initio exon-intron definitions. These data indicate that the RNA-seq-based annotation greatly improves the accuracy of exon boundaries and overall gene definitions.

To determine the false positive rate, we also compared the exon structure accuracy by calculating the exon coverage rate by mRNA short reads (Figure 3B). An exon with 99% or more coverage was defined as perfect support, 70-99% coverage as fair support, and less than 70% coverage as poor support. 95.2% of exons defined by our optimized gene model pipeline show perfect support by mRNA short reads, compared to 89.8% of exons defined by in the ab initio predictions. Only 0.8% of exons in our gene models had poor support compared to 2.8% of exons in the in ab initio gene prediction set. Thus, the gene models derived from our optimized pipeline are more supported by experimental evidence and thus have more accurate structures.

As further validation of the gene structures defined by RNA-seq, we sequenced the mRNAs for some genes with discrepant structures between the RNA-seq-derived gene set and the ab initio predictions (Figure 3C-E). For each selected gene, a cDNA spanning all exons was generated by RT-PCR and the amplicon was sequenced to provide nucleotide-level validation of the gene structure. We resolved differences in gene structure predictions for the *MFS5* gene, which encodes a predicted major facilitator superfamily membrane transporter and two hypothetical genes (i.e., genes without recognizable functions), *HYP12* and *HYP13*. The sequence of the *MFS5* cDNA shows the *MFS5* gene is composed of 6 exons, which completely matches the RNA-seq-derived structure (Figure 3C). The ab inito *MFS5* gene prediction has 5 exons, inaccurately missing the intron between exons 2 and 3. For the *HYP12* gene, 5 exons were correctly defined by RNA-seq, but the ab initio predictions missed exon 2, instead including an abnormally large 451 base-pair intron (Figure 3D). For the *HYP13* gene, RNA-seq accurately defined the gene with 2 exons whereas ab initio mispredicted the location of the second exon and added a third exon further downstream (Figure 3E). Together, these data demonstrate the greater experimental support and the improved accuracy in gene structure definition from the RNA-seq based transcriptome compared to the ab initio predicted gene models.

### Quantitative gene expression profiling

The transcription profile and relative gene expression levels for genes expressed by G186A and G217B *Histoplasma* cells were determined by counting the number of matching RNA-seq reads from each strain. Relative expression levels were calculated using the Cufflinks algorithm [43]. To enable cross-species and cross-phase comparisons, gene expression levels were normalized and calculated as Fragments Per Kilobase of exon per Million fragments mapped (FPKM; [44]). Analysis of the FPKM values for the biological replicates of each phase for each *Histoplasma* strain shows the replicates are highly similar (Additional file 1: Figure S1). Thus, the mean FPKM between replicate libraries was used for calculation of the fold change in gene expression levels.

**Figure 4:**
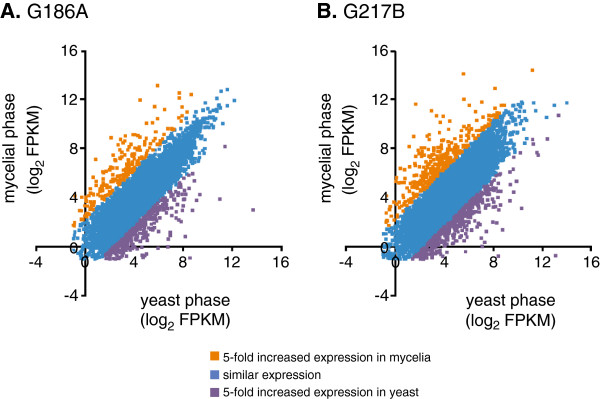
**Identification of G186A and G217B genes with enriched expression in the two different morphological phases.** FPKM values were used to compare the gene expression levels between the yeast-phase and the mycelial-phase of G186A **(A)** or G217B **(B)***Histoplasma*. Data points represent the log_2_ transformation of the FPKM value for individual gene expression in yeasts (x-axis) and mycelia (y-axis). Colors represent genes without significant phase-dependent regulation (blue; genes showing less than 5-fold change in expression), genes with increased expression in yeast-phase *Histoplasma* (purple), and genes with increased expression in mycelial-phase *Histoplasma* (orange).

General features of the gene expression levels for each strain and phase are presented in Table 2. The mean gene FPKMs for yeast and mycelia libraries was compared to determine the degree to which genes are differentially regulated between yeast and mycelial phases (Figure 4). A 5-fold difference in FPKM values was used as a conservative criterion for significant differential expression. In G186A, 534 genes are differentially regulated between phases (317 genes upregulated in yeasts and 217 genes upregulated in mycelia; Figure 4A). G217B gene expression analysis shows a similar trend: 751 genes are differentially expressed with 423 genes upregulated in yeasts and 328 genes upregulated in mycelia; Figure 4B). Overall, 6% to 9% of *Histoplasma* genes show phase-dependent expression. Gene expression data for G186A and G217B are presented in Additional file 2: Table S1 and Additional file 3: Table S2, respectively.

**Table 2:** G186A and G217B gene expression statistics

	**Yeast**		**Mycelia**	
	**G186A**	**G217B**	**G186A**	**G217B**
**FPKM range**	0 – 13514	0 – 15819	0 – 6727	0 – 17942
**FPKM median**	28	27	25	21
**Genes comprising 50% of total transcripts**	870	737	671	360

Examination of genes whose expression is upregulated in the yeast phase highlights features potentially linked to the virulence that characterizes this phase. The set of upregulated yeast-phase genes includes *CBP1*, *SOD3*, *CATB*, *CFP4*, *CFP8, ENG1, TRL1*, all well established yeast-phase expressed genes in both strains [31,45], many of which have been linked to virulence [9,10,12]. Consistently, *CBP1* is one of the most highly expressed genes (FPKM values at least 8000) and one of the most differentially expressed (at least 1000-fold increased expression in yeast compared to mycelia). The virulence-promoting *AGS1* and *YPS3* genes are also enriched in yeast, but only in G186A and G217B, respectively.

The list of genes with significant yeast-phase enriched expression (at least 5-fold compared to mycelia) is presented in Additional file 4: Table S3. In total 275 genes were upregulated in yeasts with 43 genes upregulated in both strains (100 were upregulated only in G186A yeasts compared to G186A mycelia and 132 were upregulated only in G217B yeasts compared to G217B mycelia). In G186A, the most differentially expressed gene is HC186_02213 (1735-fold induced in yeast), a gene of unknown function. In G217B, the gene most upregulated in yeast is *CBP1*. In both strains, the *GNT1* gene, which encodes an N-acetylglucosaminyltransferase involved in N-linked protein glycosylation, is consistently highly upregulated (53-fold and 78-fold in G186A and G217B, respectively). G217B yeasts also upregulate an α-mannosyltransferase *(MNN2*; 104-fold).

Genes upregulated in yeasts compared to mycelia suggests different metabolism between the phases. Genes encoding enzymes involved in coenzyme A (CoA) synthesis (2-dehydropantoate 2-reductase (*PAN5*) and pantetheine-phosphate adenylyltransferase (*CAB4*)) are also significantly upregulated in yeasts despite pantothenate in the growth medium. Various membrane transporters are also more highly expressed in yeasts compared to mycelia including *MFS*- and *ABC*-type transporters and proteins putatively transporting metabolites such as amino acids (3 transporters including the general amino acid permease *GAP1*), zinc (a zinc transporter in G186A yeasts and *ZRT2* in G217B yeasts), sulfate, and phosphate. Yeasts of both strains also upregulate the *ATG1* kinase.

Yeasts are also characterized by increased expression of various transcriptional control proteins, which may underlie the differing expression profiles of yeasts and mycelia. In G186A and G217B, a subunit of TFIIE (encoded by an ortholog of *TFA2*) is upregulated 32-fold and 73-fold, respectively. G186A yeasts and G217B yeasts compared to their respective mycelia also have higher expression of Zn-finger transcription factors (two in G186A and one in G217B). Both strains upregulate expression of the gene encoding a subunit of DNA Polymerase II, which may indicate a greater DNA synthesis capacity, is required for faster cell cycles in yeast compared to mycelia.

Although a few inferences can be made from the yeast-phase regulon, the vast majority of yeast-phase regulated genes (80% of G186A and 73% of G217B) encode hypothetical proteins with no ortholog with known biochemical or molecular function. This highlights how little is currently understood about the gene expression profiles that provide for a pathogenic lifestyle compared to non-pathogenic growth.

### Identification of strain-specific, pathogenic-phase gene expression profiles

**Figure 5:**
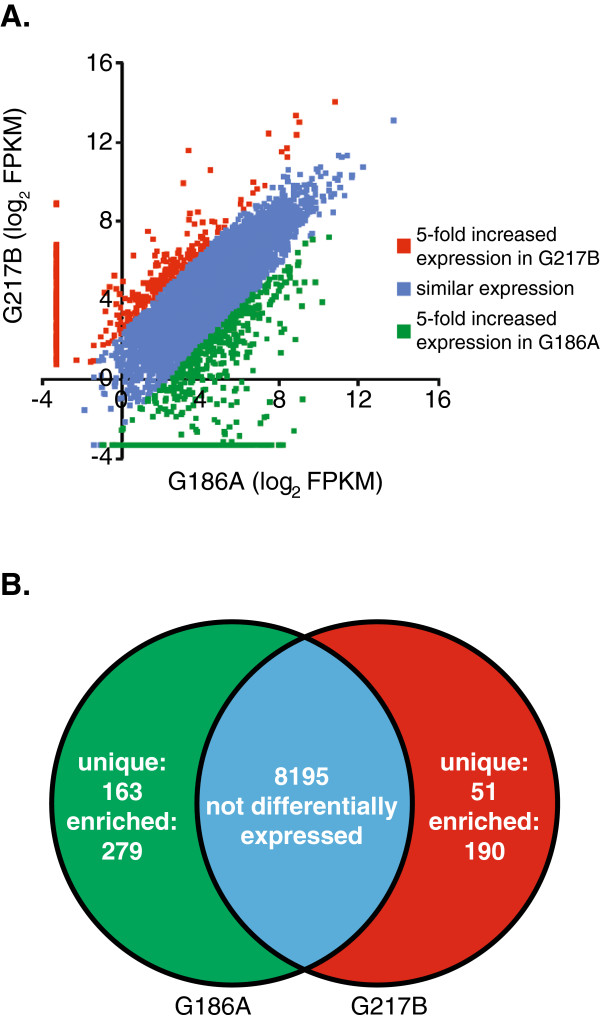
**Yeast-phase expressed genes with lineage-specific gene expression differences.** Expression levels of individual genes in G186A and G217B were compared using FPKM values for yeast-phase cells. **(A)** Data points represent the log_2_ transformation of the expression (FPKM) of individual genes in G186A yeasts (x-axis) and G271B yeasts (y-axis). Data point color indicates genes with similar expression levels in G186A compared to G217B (blue; less than 5-fold change), genes with significantly increased expression in G186A yeast (green; at least 5-fold), or genes with significantly increased expression in G217B yeast (red; at least 5-fold). Data points clustering at log_2_(FPKM) = -3.32 represent genes whose expression was FPKM < 0.1, which were then set to (FPKM = 0.1) as the lowest reasonable limit of detection. **(B)** Venn Diagram of lineage-specific gene expression: genes not differentially expressed (blue overlap) or genes with increased expression (at least 5-fold) in G186A yeast (green) or in G217B yeast (red). Genes with strain-specific expression were further divided into those that were structurally unique to the G186A or G217B genome (unique) or orthologous genes that were differentially expressed by G186A and G217B yeasts (enriched).

To discover genes that potentially contribute to virulence differences between strains, we compared the transcriptional profile of genes for the pathogenic phases of G186A and G217B. Differential gene expression by pathogenic phase yeasts includes (1) genes with higher expression in one strain (at least 5-fold difference in FPKM ratio), (2) genes only expressed by one strain (FPKM of 0 in the other strain), and (3) genes structurally unique to the genome of one strain (genes with no BLAST match to the genome of the other strain with an e-value less than 10^-35^ and covering 50% of the query gene). With inclusion of the uniquely expressed and the structurally unique genes between strains, 8978 genes in total were compared and genes with at least 5-fold differential regulation between strains identified (Figure 5A). To avoid overestimation of the fold change between backgrounds, genes with low levels of yeast phase expression (FPKM less than 0.5 in both strains) were excluded as the magnitude of the ratio of their expression ratios was unreliably magnified by low FPKM values. 100 genes overall were excluded due to low FPKM values. From this analysis, we identified 442 G186A-specific genes (279 upregulated genes and 163 structurally unique genes) and 241 G217B-specific genes (190 upregulated genes and 51 structurally unique genes) (Figure 5B).

**Figure 6:**
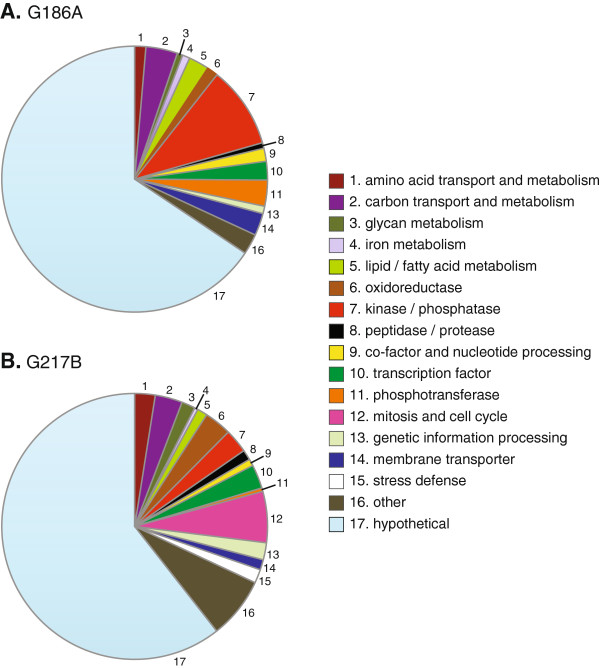
**Functional classification of G186A- and G217B- enriched genes.** Chart depicts the proportion of genes that show increased expression in G186A **(A)** or G217B **(B)** by the putative functional class of the encoded gene product. Genes encoding factors with no identifiable function based on protein sequence homology were categorized as “hypothetical.”

To identify the possible functions of the gene products of differentially expressed yeast-phase genes, protein homologies were assigned based on BLAST, Gene Ontology (GO) terms and the Kyoto Encyclopedia of Genes and Genomes (KEGG). *Histoplasma* gene products were categorized into sixteen general functional classes or were designated as “hypothetical” if no functional homology or definition could be made (Figure 6). Upregulated yeast-phase genes in G186A showed enrichments (P-value < 0.05) in 15 different classifications (Figure 6A), G217B yeast-phase upregulated genes included 17 different classifications (Figure 6B). For the majority of lineage-enriched genes, no specific function could be assigned based on the amino acid sequence. As a class, genes encoding kinase/phosphatase functions as well as membrane transport proteins are enriched in G186A yeasts compared to G217B yeasts. For G217B, genes encoding factors for glycan metabolism, oxidoreductases, and functions linked to mitosis and cell cycle progression are enriched in G217B yeasts compared to G186A yeasts. Both strains have similar number of genes encoding amino acid, carbon, and lipid metabolism, although the specific gene products in these categories differ between the strains.

The complete list of genes unique to G186A or with enhanced expression in G186A is detailed in Additional file 5: Table S4. Genes structurally unique to G186A or only expressed by G186A yeasts comprise most of the enriched kinase/phosphatase functions but also include *FET3*, *FTR1*, an *ARN*-family siderophore transporter (*ARN2*), and a predicted quinone oxidoreductase (*ZTA1*). Among the genes with enriched expression in G186A yeasts compared to G217B are those encoding a glycosyl transferase (*ATG26*) and a chitinase (*CHI1*), two phospholipases (*PLB1* and *PLD1*), a glyoxylate reductase (*GOR1*), and an N-acetyltransferase (*ARD1*).

In contrast, G217B yeast has unique or enriched expression of a different set of genes that are detailed in Additional file 6: Table S5. Expressed genes unique to the G217B genome, and for which clear orthologous proteins could be identified, encode a putative beta-glucanase (*TOS1*) and two kinases potentially involved in cellular signaling (*PKH2* and *GSK3*). A v-SNARE protein is also uniquely encoded in the G217B genome by the *YKT6* gene, which may indicate differential trafficking of vesicles in this strain. Genes of the siderophore biosynthesis gene cluster (i.e., *SID3*, *SID4*, *OXR1*, *MFS1*, and *NPS1*); [46] are preferentially expressed by G217B yeasts compared to G186A yeasts even though both genomes have this cluster. Interestingly, genes encoding histones (*H1*, *H2A*, *H2B*, *H3*, and *H4*) all show increased expression in G217B yeasts. Genes encoding a copper transporter (*CTR3*) and one of three carbonic anhydrases (*CAH1*) are 68-fold and 30-fold more expressed, respectively, in G217B than G186A. The genes for a ras-GTPase activating protein (*IQG1*), a MAP kinase kinase kinase (*SSK2*), a Ca^2+^/calcineurin-dependent transcription factor (*CRZ1*), and a histone deactylase (*HOS1*) show increased expression in G217B. G217B-enriched genes also include factors linked to *Histoplasma* virulence, namely Yps3 (nearly 300-fold), but also the secreted superoxide dismutase (Sod3; [10]) and the secreted catalase (CatB; [12]).

**Figure 7:**
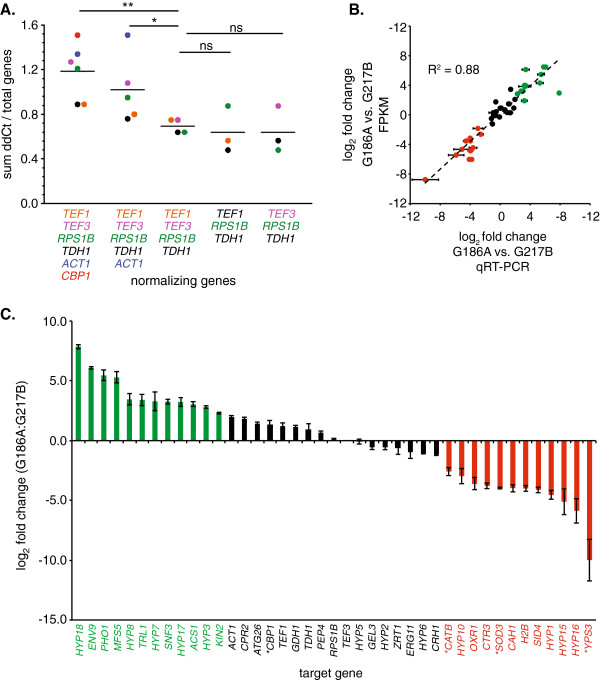
**Validation of FPKM-based gene expression by quantitative RT-PCR. (A)** Identification of genes with minimal variation in expression between strains. Gene expression of candidate normalization genes (*TEF1*, *TEF3*, *RPS1B*, *TDH1*, *ACT1*, and *CBP1*) was determined by qRT-PCR. Expression values were normalized to one member of the gene set and the total difference in cycle threshold between strains (y-axis) determined for the remaining genes. Colors correspond to which gene was used for normalization and the resultant total variation in cycle thresholds. The process was repeated iteratively by removing the gene, which when used as the normalizer, resulted in the greatest overall difference in expression between strains. Included genes are listed under the x-axis. Horizontal bars represent averages. Significantly different total cycle threshold variations determined by Student’s *t*-test are indicated (*, P < 0.5; **, P < 0.1; ns, non-significant). **(B)** Correlation between FPKM and qRT-PCR determination of gene expression between G186A and G217B yeasts. Data points represent the log_2_-transformed value of the fold-change in expression determined by qRT-PCR after normalization to *TEF3* (x-axis) or by FPKM ratio (y-axis). Data point color indicates genes with no difference in expression (black; < 5-fold), genes upregulated in G186A (green; > 5-fold), and genes upregulated in G217B (red, > 5-fold). Error bars represent the standard deviation of the relative expression of three replicate yeast cultures for each strain. **(C)** Relative expression of selected genes in G186A yeasts compared to G217B yeasts. Bars represent the average fold change (log_2_) and error bars represent the standard deviation (n = 3 for each strain). Analyzed genes (x-axis) and data are colored to indicate genes with no enriched expression in either strain (black), genes upregulated in G186A yeasts (green; > 5-fold), and genes upregulated in G217B yeasts (red; > 5-fold). Asterisks denote genes with established roles in *Histoplasma* yeast virulence.

To validate the pathogenic-phase gene expression differences between strains, we used quantitative RT-PCR as an independent determination of yeast gene mRNA levels. Accurate quantification between strains requires normalization to genes whose expression does not significantly vary between strain backgrounds. A number of housekeeping genes have been used in intraspecies expression quantifications, but few have addressed the appropriate normalizer for interspecies comparisons. To identify genes with minimal variation between strains, we examined a set of constitutively expressed and housekeeping genes representing translation (ribosomal subunit and translation elongation factors; *RPS1B*, *TEF1*, and *TEF3*), metabolism (glyceraldehyde-3-phosphate dehydrogenase; *TDH1*), the cytoskeleton (actin; *ACT1*), and yeast-phase growth (*CBP1*) and determined the degree of co-variation with each other between strains. The relative expression of each gene was computed using the ΔΔ*C*_*T*_ method [47] after normalization to each one of the other genes. As an indicator of overall transcriptional discrepancy among these genes, the magnitudes of the ΔΔ*C*_*T*_ values obtained were summed and the normalizing gene that produced the greatest amount of overall interstrain variation was identified (Figure 7A). This process was repeated iteratively after excluding this normalizing factor from subsequent calculation until no significant change in the total variation was reached by further normalizing gene exclusion. Through this analysis, we determined that *CBP1* and *ACT1* were more variable than desired between strains and therefore unsuitable as normalizing factors, and that *TEF1*, *TEF3*, *TDH1*, and *RPS1B* were expressed at sufficiently similar levels between strains to be used as normalizing genes for quantitative RT-PCR (Figure 7A). The *TEF3* gene was selected for normalization of relative expression levels.

Quantitative RT-PCR was used to confirm the expression differences between strains for a subset of 41 differentially expressed and similarly expressed genes. Both genes encoding proteins with recognizable functions as well as genes without known functions (*HYP* genes) were included. In addition the *CBP1*, *SOD3*, *CATB*, and *YPS3* genes were included to determine if expression differences in these known virulence factors [9,10,12,23] correlated with differences in strain virulence. The relative fold-change in expression between strains determined by FPKM analysis was highly correlated with differences determined by qRT-PCR (R^2^ = 0.88; Figure 7B) providing validation of the FPKM analysis. Genes significantly more expressed by G186A included *HYP18* (234.5-fold), *ENV9* (68.0-fold), a putative alkaline phosphatase (*PHO1*; 43.9-fold), *MFS5* (39.2-fold), *HYP8* (11.0-fold), *TRL1* (10.6-fold), *HYP7* (9.8-fold), *SNF3* (9.6-fold), *HYP17* (9.4-fold), *ACS1* (8.2-fold), *HYP3* (6.9-fold), and *KIN2* (4.9-fold). Genes significantly more expressed by G217B included *YPS3* (1019.3-fold), *HYP16* (58.4-fold), *HYP15* (34.4-fold), *HYP1* (23.4-fold), *SID4* (17.4-fold), *H2B* (16.0-fold), *CAH1* (15.7-fold), *SOD3* (15.7-fold), *CTR3* (13.9-fold), *OXR1* (12.3-fold), *HYP10* (7.8-fold), and *CATB* (6-fold) (Figure 7C). Regarding known virulence factors, *YPS3*, *SOD3*, and *CATB* were more highly expressed by G217B while *CBP1* was slightly more expressed in G186A.

### Factors determining inter-strain variation in expression of genes

**Figure 8:**
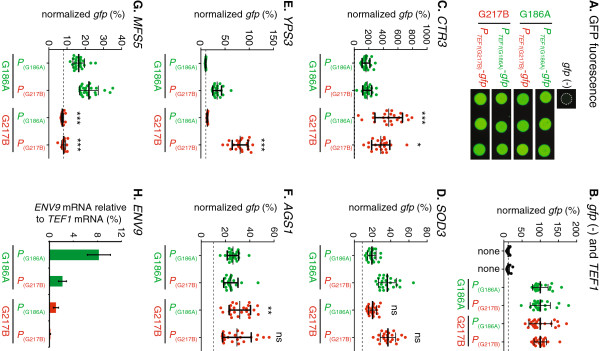
**Regulation of differentially expressed genes by cis- or trans-acting factors.** Promoters of genes differentially expressed in G186A and G217B yeasts were fused to a *gfp* reporter and transformed into both strain backgrounds. Promoter regions (“*P*”) were amplified from G186A or G217B genomic DNA and the constructs transformed into *Histoplasma* yeasts. Expression of the *gfp* reporter was measured in transformants by quantifying GFP fluorescence **(A**-**G)** or by qRT-PCR measurement of *gfp* mRNA **(H)**. **(A)** Representative images of GFP fluorescence of individual transformants in which *gfp* expression was driven by the G186A and G217B *TEF*1 promoters (*P*_*TEF1*_) in the G186A (green) or G217B (red) background. **(B**-**H)** Data represents the GFP fluorescence from individual transcriptional-reporter gene fusion transformants in the G186A background (green data points) and the G217B background (red data points). Data is normalized to *gfp* expression driven by the *TEF1* promoter to enable interspecies comparisons. Dashed horizontal lines indicate the autofluorescence of G186A and G217B yeasts lacking the *gfp* reporter (“none”). Reporter gene expression driven by the *TEF1* promoter **(B)**, the *CTR3* promoter **(C)**, the *SOD3* promoter **(D)**, the *YPS3* promoter **(E)**, the *AGS1* promoter **(F)**, the *MFS5* promoter **(G)**, and the *ENV9***(H)** was measured. The *AGS1* promoter from G217B was repaired by removal of the inserted repetitive DNA for comparison to the native G186A promoter. Horizontal bars represent means ± standard deviations (n ≥ 22 **(A**-**G)** or 3 pools of 3 replicates each in **(H)**). Significant differences in expression between the G186A and G217B genetic backgrounds were determined by Student’s *t*-test and are indicated by asterisks over the G217B transformant data (*, P < 0.05; **, P < 0.01, ***; P < 0.001; ns, non-significant).

Differential gene expression is often regulated at the level of transcription, which is influenced by cis- and trans-acting regulatory factors. To determine if cis- or trans-acting factor differences between strains determine *Histoplasma* interstrain gene expression variability, we created transcriptional reporter fusions using putative promoter regions upstream of differentially expressed genes and transformed them into the G186A and G217B backgrounds. Promoter regions (0.6 to 2 kb of sequence upstream of the CDS) for *TEF1*, *CTR3*, *SOD3*, *AGS1*, *YPS3*, *MFS5* and *ENV9* from both G186A and G217B were fused to a *gfp* reporter gene. To test if cis-acting factors (e.g., promoter sequence polymorphisms between strains) or trans-acting factors (e.g., transcription factors differences) controlled the differential transcription, promoter fusions for each of the G186A and G217B promoters were transformed into both the G186A-background. The level of GFP fluorescence in transformed colonies was used as a surrogate measure of gene expression levels. Consistent with the RNA-seq and qRT-PCR analyses, *TEF1* promoter fusions to gfp yielded similar fluorescence regardless of the strain from which the promoter was derived or the background into which it was placed (Figure 8A). Therefore the fluorescence of the *TEF1* promoter fusions were used to normalize exposure times for GFP fluorescence between backgrounds for the other promoter tests.

For genes more highly expressed in G217B, we tested whether the promoter sequence was responsible for the variation in gene expression. Three genes more highly expressed in G217B were selected for investigation: *CTR3*, *SOD3*, and *YPS3*. For *CTR3*, 1.45 kb of upstream sequence was sufficient to cause expression of the *gfp* reporter (Figure 8B). The *CTR3* promoter nucleotide sequences are 92% identical between G217B and G186A. GFP reporter fluorescence was significantly higher in the G217B background (2.1 to 2.9-fold) regardless of the strain from which the promoter sequence originated. This indicates that the enhanced expression of *CTR3* in G217B is independent of polymorphisms in the promoter but highly dependent on the genetic background (i.e., trans-acting factors that differ between the strains). In contrast to *CTR3*, cis-acting factors (i.e., promoter sequences) are the major determinants of interstrain expression differences for *SOD3* and *YPS3*. The G217B sequence of the *SOD3* promoter (1.9 kb) provided higher transcription of the GFP reporter than the G186A *SOD3* promoter in both strain backgrounds (1.8-fold and 1.9-fold; Figure 8C). For regulation of *YPS3* expression, the G186A sequence of the *YPS3* promoter (1.9 kb of upstream sequence) could not drive transcription of the GFP reporter in either strain background whereas the G217B sequence of the promoter enabled transcription of the reporter gene (Figure 8D). Thus, transcription of *YPS3* is controlled primarily by the promoter sequence, although transcription of the reporter was 2.4-fold higher in the G217B background than the G186A background suggesting the contribution of some additional trans-acting factors operating in G217B.

To determine whether cis- or trans-acting factors determined expression differences of genes more highly expressed in G186A, promoter fusions were created using sequences upstream of the *AGS1* (1.9 kb), *MFS5* (1.9 kb), and *ENV9* (1.9 kb) genes. Previously we showed that *AGS1* transcription was significantly attenuated in G217B due to insertional disruption of the promoter [15] indicating cis-control of *AGS1*. Removal of the disrupting sequences from the G217B *AGS1* promoter restored transcriptional activity in both G186A and G217B backgrounds indicating deficient *AGS1* expression in G217B is primarily due to the disruption in the promoter (Figure 8E). For *MFS5* promoter-*gfp* fusions, transcription of the reporter gene was strongly influenced by the strain background with both G186A and G217B *MFS5* promoter sequences driving 2.0-fold to 3.0-fold more transcription in the G186A background (Figure 8F). Thus, *MFS5* transcriptional differences arise predominantly from differences in the strain background rather than promoter polymorphisms. Transcriptional *gfp* fusions to the *ENV9* promoter (up to 1.9 kb) failed to produce any GFP fluorescence in transformants irrespective of the genetic background or which promoter sequence was used. We suspect that the lack of GFP protein production results from unknown alternative translational start sites, which could shift *gfp* translation out of frame. As an alternative means of testing for transcriptional activity of the different *ENV9* promoters without requiring reporter protein synthesis, we used quantitative RT-PCR of the *gfp* transgene. For *ENV9*, both the promoter and background influenced the expression of the transgene with increased expression driven by the G186A promoter. This indicates that *ENV9* expression is strongly influenced by promoter sequence, but the strongest expression was in the G186A background indicating trans-acting factors also contribute to *ENV9* expression differences (Figure 8G). Together, these data provide examples that show gene expression differences among *Histoplasma* strains that are determined by cis acting factors (i.e., promoter sequence) and/or variations in trans acting factors (e.g., translation factor abundance or activity) that exist between strain backgrounds.

## Discussion

While genome sequencing provides an excellent starting point for characterization and analysis of *Histoplasma capsulatum*’s nearly 10,000 genes, accurate gene structure determination requires experimental evidence. Use of tiling microarrays with isolated RNAs improved gene structure definition in *Histoplasma*[27]. In this study, we used RNA-seq to inform gene models for two *Histoplasma* clinical isolates, G186A and G217B, representing divergent phylogenetic clades. In addition to spliced-alignment of the mRNAs to a references genome, we used de novo assembly of the mRNAs at multiple points of the annotation pipeline to further refine annotations with experimental evidence. The assembled mRNA sequences generated more precise gene models than those derived from ab initio predictions, and improved the evidence-based resolution of the gene structures to the nucleotide level.

In addition, RNA-seq enabled a comparative gene expression approach to identify phase-specific as well as strain-specific gene profiles. As *Histoplasma* cells exist as yeasts during mammalian infection, we directed our efforts at identification of genes preferentially expressed by these virulent cells compared to mycelia that are unable to establish disease. Overall, we found that 6% to 9% of the genome is differentially expressed between these two phases. Microarray-based studies found 5% [26] to 19% [29] of genes had phase-dependent regulation. The dramatically increased percentage of regulated genes in Inglis et al., likely results from the 3-fold differential expression criteria they used, whereas we required a more stringent threshold of 5-fold differential expression. By RNA-seq, we found that growth as yeast cells results in induced expression of 3% to 5% of the genes. Assuming that increased expression confers the characteristics required for pathogenic-phase growth, 300 to 400 genes could potentially contribute to *Histoplasma* virulence.

Genes preferentially expressed by yeast-phase cells of both strains suggest some functions that characterize pathogenic-phase growth. Yeast cells upregulate gene expression of enzymes for glycosylation of proteins (Gnt1 and an α-mannosyltransferase). These yeast-phase-expressed glycoysltransferases suggest that yeasts, but not mycelia, rely upon glycosylation of extracellular proteins as they transit through the ER and Golgi. In support of this, Holbrook et al. found that proteins from yeast culture filtrates, but not mycelia culture filtrates, are heavily glycosylated [45]. Yeast cells also upregulate enzymes involved in CoA synthesis (e.g., Pan5 and Cab4) that may indicate yeast have additional CoA/acetyl-CoA need for carbon and lipid metabolism pathways. Yeast cells have increased expression of *ATG1*, a kinase that is involved in regulating autophagy in response to environmental signals [48-50]. As the cDNA libraries were prepared from yeasts and mycelia grown in identical growth medium, the yeast-phase upregulated genes presumably represent gene expressions regulated by lifestyle rather than nutrient availability.

The phylogenetic groups represented by G186A and G217B strains are notably dissimilar in many of the yeast-phase-enriched genes. Only 43 of the 275 genes differentially expressed by yeasts compared to mycelia are common between the two strains. This may not be surprising as G186A and G217B have been shown to have different relative virulence in murine models [15-17] and utilize different virulence factors to mediate their pathogenesis [15,19,23]. Gene expression differences between strains indicate that the different *Histoplasma* lineages are more dissimilar than previously assumed. For example, expression of a high affinity copper transporter (*CTR3*) and a carbonic anhydrase (*CAH1*) are substantially increased in G217B while an MFS-family transporter (*MFS5*) shows 100-fold more expression in G186A. Our data indicate that the differential regulation of genes between strains results from differences in cis-acting sequence polymorphisms in the promoter of genes as well as trans-acting factors that reflect the different background of the strains.

Our expression profiling highlights differences in iron acquisition between G186A and G217B. We found that the *SID1* gene, which is involved in siderophore synthesis, is only yeast-phase enriched in G217B contrary to the microarray analysis [29]. Both G186A and G217B express *ARN*-family siderophore transporters. In G217B, one family member has enriched expression in yeasts compared to mycelia. On the other hand, G186A yeasts express an ARN-family transporter unique to the G186A genome and the G186A-specific iron oxidase and permease encoding genes *FET3* and *FTR1*. This data indicates G217B and G186A differ in the mechanisms for iron acquisition.

The G186A-enriched gene set suggests G186A yeasts differ from G217B yeasts in lipid metabolism. G186A has increased expression of two phospholipases, *Plb1* and *Pld1*, as well as the acyl chain desaturase *Ole1*, which has been linked to *Histoplasma* thermotolerance [51,52]. Together, these factors may suggest that G186A is better able to remodel lipids to adapt to thermal stresses encountered during infection. G186A also preferentially expresses a number of kinases (a *SKY1*-related kinase and a *HOG1*-related kinase) that may help it sense and respond to conditions related to infection of phagocytes.

The cell walls of G186A and G217B yeasts are biochemically different from each other. Most notable is the α-glucan difference between strains. Our expression analysis highlights additional glycan-related enzymes that have differential expression between the strains. G186A yeasts express higher levels of the *CHI1* gene encoding a chitinase and a glucosyl transferase (*ATG26*). On the other hand, G217B yeasts have increased expression of the *TOS1* gene and a putative exo-glucanase gene (*EXG2*). The Tos1 factor is a putative β-1,3-glucanase [53,54] that responds to cell stress [55]. Cell wall modification by Tos1 and/or Exg2 may be essential for modifying the yeast cell wall of G217B, which lacks the virulence-promoting α-glucan of G186A yeasts. Thus, the surface composition differences between these two lineages may extend beyond the α-glucan content and this may directly affect how G186A and G217B yeasts interface with host cells.

Compared to G186A, G217B cells express higher levels of oxidative stress defense genes. G217B yeasts show higher transcription of the extracellular catalase (*CATB*) and the extracellular superoxide dismutase (*SOD3*) genes, the products of which are required for *Histoplasma* virulence [10,12]. This may indicate G217B yeasts may survive the phagocyte oxidative burst better than G186A yeasts. Together these data suggest G217B may rely on enhanced defense mechanisms rather than evasion of phagocyte detection through modification of the glycan composition of the cell wall.

Although inferences and hypotheses based on differentially expressed genes can be made, the majority of differentially expressed genes encode proteins with no known function, whether comparing pathogenic and non-pathogenic phases or comparing between strains. This underscores how little we understand the biology of *Histoplasma* and the need for functional studies. The experimental evidence-based transcriptomes established in this study will provide an important framework for identifying genes that underlie the pathogenesis differences between phases of this dimorphic fungal pathogen.

## Conclusions

In this study, we used mRNA sequencing (i.e., RNA-seq) to refine gene models for two *Histoplasma* clinical isolates, G186A and G217B, representing divergent phylogenetic clades with different virulence. Quantitation of the transcriptional profiles identified phase-specific and strain-specific expression differences that correlate with differences in fungal virulence. Depending on the strain, between 6% and 9% of genes are differentially regulated between the virulent yeast and avirulent mycelial phases. As the gene content is equivalent between yeasts and mycelia of a given strain, these findings underscore the fact that *Histoplasma* pathogenesis is primarily a function of gene expression differences between the dimorphic phases.

## Methods

### *Histoplasma* strains and growth conditions

The wild-type *Histoplasma* strains used were the clinical isolates G186A (ATCC 26029) and G217B (ATCC 26032). Uracil auxotroph strains for transformation with URA5-based plasmids were the *ura5*-deletion strains OSU1 and WU15, which were derived from the G186A and G217B wild-type isolates, respectively [8,56]. Yeast and mycelial-phase fungal cells were cultured in *Histoplasma*-macrophage media (HMM) [57]. For growth of uracil auxotrophs, HMM was supplemented with uracil (100 μg/ml). For growth on plates, HMM was solidified with agarose and supplemented with 25 μM FeSO_4_. To maintain yeast-phase morphology, yeasts were cultured at 37°C with agitation. Mycelia cultures were grown at 25°C.

### RNA isolation

Yeasts grown to late exponential phase (approximately 72 hours) were collected by centrifugation, resuspended in RNAlater (Ambion), and frozen at -80°C. To collect mycelia, hyphae were separated from the culture media by filtration through Whatman #5 filter paper and the mycelial cells placed in RNAlater and frozen at -80°C. Total RNA was isolated using the RiboPure-Yeast Kit (Ambion) using mechanical disruption of fungal cells and purification of RNA from the lysate on an RNA-binding column. RNA quality was assessed with the Bioanalyzer platform (Agilent). Two biological replicate cultures of yeast and mycelia were prepared for transcriptome libraries.

### Library preparation and transcriptome sequencing

Library preparation and sequencing of mRNAs by RNA-seq were performed at the Molecular and Cellular Imaging Center at the Ohio Agriculture Research and Development Center at Ohio State University. cDNA libraries were prepared using the TruSeq RNA sample preparation kit (Illumina). Briefly, mRNA was isolated from total RNA using poly-A capture. The mRNAs were fragmented by cation treatment with heat and then converted to cDNA by reverse transcription and second strand synthesis. cDNA ends were repaired and adenylated to facilitate ligation of indexed adaptors. Following ligation of adaptors, 15 cycles of PCR were performed to enrich for cDNA fragments with adaptors on both ends of the molecule. Indexed libraries were pooled and sequenced using the Illumina GAII platform to generate paired-end reads. Library reads were deconvolved post-sequencing by virtue of the adaptor index on each cDNA molecule.

### Gene modeling and annotation

The spliced alignment tool Tophat [36] was used to align short reads from G186A and G217B mRNAs to the to the G186A reference genome (Broad Institute). Strict parameters were used (0 mismatches and no gaps) for mapping G186A reads to generate a highly accurate alignment result. Alignments were used to indicate the transcript regions and identify splice junctions, which were subsequently used as hints to derive gene structure models with Augustus [37,38]. Augustus was also used to produce a gene prediction set for additional sensitivity. In parallel, Inchworm [39] was used for de novo transcript assembly of RNA-seq short reads and previously sequenced EST reads. BestORF (Molquest package, Softberry) was used to identify open reading frames in the de novo transcript assembly. The Tophat/Augustus gene models were refined by the de novo transcript-based gene structure evidence using the PASA algorithm [40]. The three data sets were integrated by using the spliced-alignment model as a base and adding in unique genes (based on genomic locations) from the other two data sets. Manual inspection and refinement of the gene structures was included to divide likely gene fusions (genes with unusually large introns, i.e., introns > 350 base pairs) or to combine potentially split genes (genes separated by unusually small intergenic distances, i.e., < 500 base pairs). Repetitive genes were identified in the final gene set by BLAST search against genome sequences and identification of those genes with two or more matches to the genome with at least 50% coverage or an E-value less than 10^-30^. To identify splicing signals, 15 bp of both ends of all introns in the G186A gene models were extracted and fed into the MEME motif finder [41]. Functional annotations of genes were assigned to the G186A and G217 gene sets using Blast2GO [58], KAAS [59], and BLAST searches of NCBI protein databases to identify homologous genes and/or protein functions. Reciprocal top-hit BLAST was used to assign orthologous identities between strains.

### Gene expression analysis of RNA-seq data

For RNA-seq-based expression analysis, the normalized gene expression (FPKM) for each defined gene was calculated by Cufflinks and Cuffdiff [43]. For these analyses, repetitive genes or genes with extremely low expression in all libraries (FPKM less than 0.1) were excluded. For cross-species gene expression comparisons, the G186A gene set was used as the reference gene models for counting reads. G217B mRNA reads were matched to the G186A gene set by allowing for 6 mismatches (which maintains 92% nucleotide sequence identity between strains). G217B mRNA reads that did not match any G186A gene model were extracted and assembled de novo into transcripts using Inchworm [39] and the genes designated as G217B unique genes. The common genes between strains were then compared using Cuffdiff to identify those genes with significantly different expression (at least five fold, q value < 0.01) between G186A and G217B. To avoid artificially high ratios of expression due to very low expression in one strain, all FPKM values less than 0.5 were set to 0.5 before ratios of expression were calculated. The final set of genes with differential expression was subsequently filtered to remove those genes with low expression (FPKM less than 0.5 in both strains). The differentially expressed gene sets were then combined with the structurally unique genes of each strain to generate the final set of genes with strain-specific expression.

### Endpoint and quantitative RT-PCR

Three micrograms of total RNA was reverse transcribed using SuperScript III reverse transcriptase (Invitrogen) and 15-mer Oligo (dT) primers and genomic DNA removed by DNAse treatment. For endpoint RT-PCR, reaction mixes included 0.5 μM gene-specific primers, 0.2 mM dNTPs, and 1:10 dilution of the reverse-transcribed RNA. Reactions using RNA in the absence of reverse transcription were performed to verify the lack of genomic DNA in RNA isolations. For quantitative PCR, reverse-transcribed RNA templates were used at a 1:10 concentrations in a PCR master mix with SYBR green (Bio-Rad) and 0.5 μM each gene-specific primer (Additional file 7: Table S6). PCR products were amplified for 30 cycles at 94°C for 10 seconds, 52°C -55°C for 15 seconds, and 68°C for 30 seconds using a realplex^2^ thermal cycler (Eppendorf). Cycle threshold (CT values) were calculated with the realplex software (v2.2) using the CalQplex algorithm (Eppendorf). Transcript levels were normalized to the *TEF3* gene. Relative fold changes in gene expression between strains were calculated using the ΔΔ*C*_*T*_ method [47]. For determination of *ENV9* promoter activity, RNA was isolated from nine independent transformants containing the *P*_*ENV9*_*-gfp* transcriptional reporter fusion and the RNA was reverse transcribed as above. Transcriptional activity of the reporter was determined by qPCR of the *gfp* gene using *gfp*-specific primers and the relative *gfp* mRNA abundance compared to *TEF1* after normalization of all *C*_*T*_ values to the *TEF3* gene [60].

### Analysis of promoter activity

Promoter activity was determined by creation of transcriptional fusions to a gfp reporter gene. Promoters were amplified with Phusion High-Fidelity Polymerase (NEB) and cloned into plasmids pCR623 or pCR628 that contain the *gfp* reporter. Putative promoter regions encompassed sequences upstream of the start codon from G186A and G217B for the *TEF1* (661 bp; pCR640 and pCR639), *YPS3* (1873 bp; pMK43 and pMK42), *SOD3* (1951 bp; pMK49 and pMK48), *CTR3* (1454 bp; pMK33 and pMK32), *AGS1* (1916 bp; pCR637 and pCR635), *MFS5* (1943 bp; pMK51 and pMK52), and *ENV9* (1961 bp; pMK47 and pMK46) genes. Promoter constructs were sequenced and then transformed into *Histoplasma* OSU1 or WU15 using *Agrobacterium tumefaciens*-mediated transformation [61]. Ura^+^ transformants were selected by plating on HMM media with 10 μg/mL tetracycline at 37°C. Individual transformant colonies were picked and spotted onto HMM medium and the GFP fluorescence of individual spots quantified using an AlphaImager UV transillumination system (CellBiosciences: [15]. GFP fluorescence measurements were all normalized to GFP expression levels of transformants with GFP under control of the *TEF1* promoter.

### Supporting data

Short reads of *Histoplasma* mRNAs generated by Illumina sequencing have been submitted to the NCBI Sequence Read Archive as accession numbers SRX332607 (G186A yeast), SRX332751 (G186A mycelia), SRX332749 (G217B yeast), and SRX332752 (G217B mycelia). Assembled transcripts and FPKM expression values are available at http://microbiology.osu.edu/RappleyeHistoplasma and the Additional files.

## Competing interests

JAE, MMK, CC, JH, TKM, and CAR are not affiliated with commercial entities and have no financial conflicts of interests.

## Authors’ contributions

JAE and CAR conceived and designed the study. JAE, MMK, and CAR prepared the samples and performed the experiments. CC analyzed the sequencing data and performed the bioinformatic studies. JH and TKM participated in the sequence data analysis. JAE and CAR drafted the manuscript. All authors read and approved the final manuscript.

## Supplementary Material

Additional file 1: Figure S1 Correlation of gene expression levels between biological replicate yeast and mycelial samples. Gene expression levels (FPKM values) were determined for G186A (A) and G217B (B). Data represents the FPKM value for individual gene expression in two biological replicated samples of yeast mRNA (left panels) and mycelial mRNA (right panels). Correlation between samples (R2) is indicated on each graph. Diagonal line represents equivalent expression between samples. Click here for file

Additional file 2: Table S1 Expression of G186A genes. Click here for file

Additional file 3: Table S2 Expression of G217B genes. Click here for file

Additional file 4: Table S3 Genes with enriched expression in the yeast phase. Click here for file

Additional file 5: Table S4 Genes with enriched expression in G186A yeasts. Click here for file

Additional file 6: Table S5 Genes with enriched expression in G217B yeasts. Click here for file

Additional file 7: Table S6 qPCR primers. Click here for file
