# Supplementary material for: Histoplasma yeast and mycelial transcriptomes reveal pathogenic-phase and lineage-specific gene expression profiles
Source: BMC Genomics. 2013 Oct 10;14:695. doi: 10.1186/1471-2164-14-695 (PMC3852720; doi:10.1186/1471-2164-14-695)
Supplement: Additional file 1: Figure S1 — Correlation of gene expression levels between biological replicate yeast and mycelial samples. Gene expression levels (FPKM values) were determined for G186A (A) and G217B (B). Data represents the FPKM value for individual gene expression in two biological replicated samples of yeast mRNA (left panels) and mycelial mRNA (right panels). Correlation between samples (R2) is indicated on each graph. Diagonal line represents equivalent expression between samples. [file 1471-2164-14-695-S1.pdf]

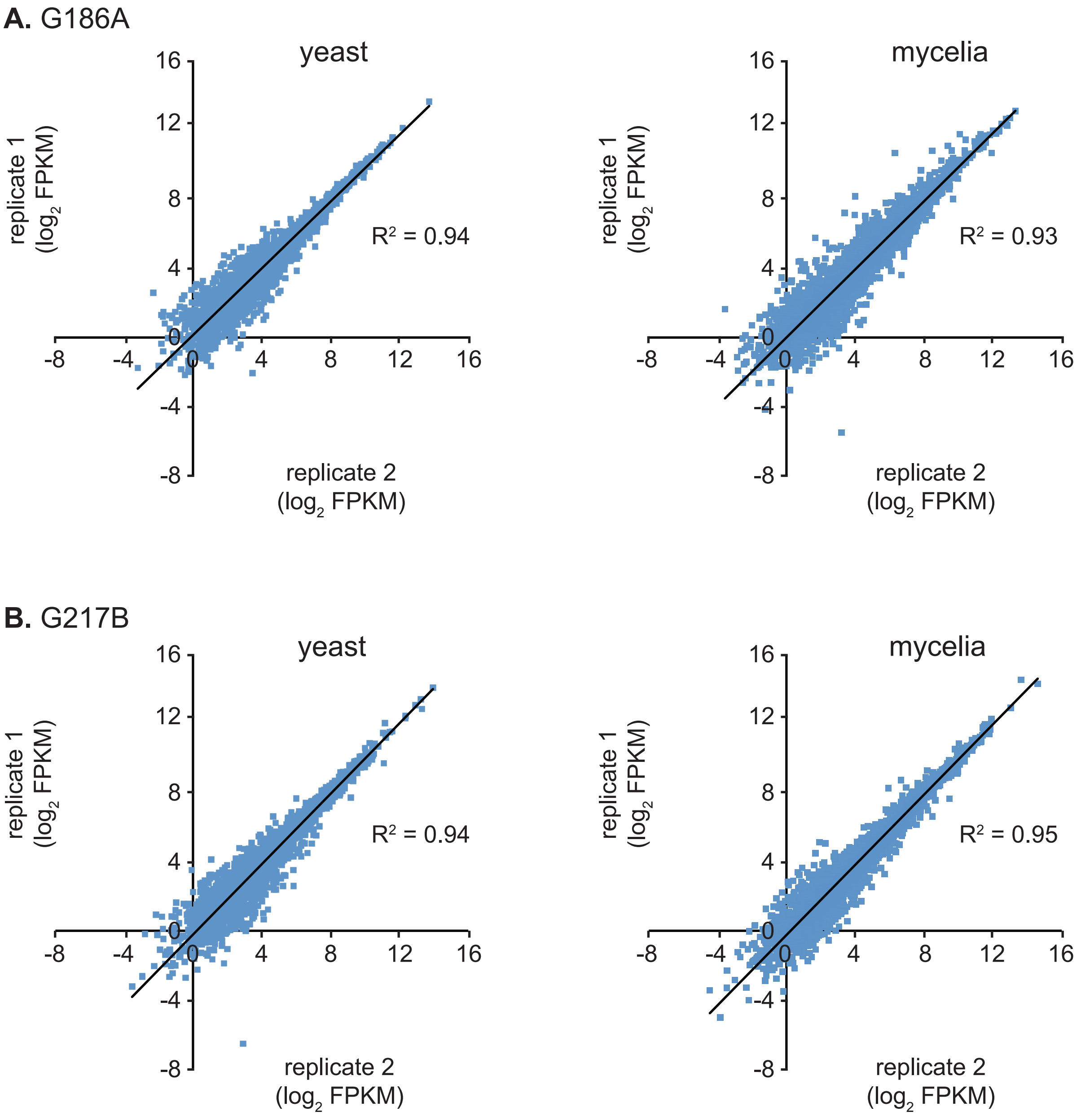

Supplementary Figure 1. Correlation of gene expression levels between biological replicate yeast and mycelial samples. Gene expression levels (FPKM values) were determined for G186A (**A**) and G217B (**B**). Data represents the FPKM value for individual gene expression in two biological replicated samples of yeast mRNA (left panels) and mycelial mRNA (right panels). Correlation between samples (R^2) is indicated on each graph. Diagonal line represents equivalent expression between samples.
