# Supplementary material for: Histoplasma yeast and mycelial transcriptomes reveal pathogenic-phase and lineage-specific gene expression profiles
Source: BMC Genomics. 2013 Oct 10;14:695. doi: 10.1186/1471-2164-14-695 (PMC3852720; doi:10.1186/1471-2164-14-695)
Supplement: Additional file 7: Table S6 — qPCR primers. [file 1471-2164-14-695-S7.doc]

**Supplemental Table 6: qPCR primers**

| **Target gene** | **F-primer sequence** | **R-primer sequence** |
| --- | --- | --- |
| *ACS1* | CGTATTTTGGGATCTGTTGGCG | ATGGCAGGCTCAATGCCGAAG |
| *ACT1* | GGTTTCGCTGGCGATGATGCTC | AAGGACGGCCTGGATGGAGACG |
| *ATG26* | CAGGATGACGCCAGCCGAAG | CAGTAACGCCATCATACCAGCCA |
| *CAH1* | GCCCATGTAATCCGCAACGCC | TAAATGTCAACATGCCACAGCCC |
| *CATB* | GAAGGCAATTTTGATATCGTTGGA | CCATCAACATGACGCATTGAGC |
| *CBP1* | GACTGGGCCGCCTGTCTCA | AGGTTGCAGTAGCAGCGCAAG |
| *CPR2* | CTATGGCGAGAAGTTTGCTGATG | CGAGAACCTCGCCGAAGACG |
| *CRH1* | GGTGATCCTTCCAATCCTCCTG | TGGGCCCGAGCCAGGAGTTC |
| *CTR3* | GGAACTGGACTGTATTGAATGCCTG | GGAGCATGACAAGAAGAATGACA |
| *ENV9* | GAAACTGCATTACCTTCGTCGC | AGACAGAGCTTTACCAACATCGC |
| *ERG11* | CGCTTTTGTAGCAGCCGTC | GCGTTCACATCTTTCAGCTTGC |
| *GDH1* | CTGTGGGAGGGCGTATTGACTG | GAAGCTTTCTTTGCCGTCCGTG |
| *GEL3* | TTACGTCAAGGCGGTCACTCG | GCAGTCCGTAACCCGAGAATAGC |
| *GFP* | GACGTAAACGGCCACAAGTTCAGC | CACGCCGTAGGTCAGGGTGGTC |
| *H2B* | TCCTACATCTACAAAGTCCTCAAGCA | GCTTGGAGGCTTCAGTGGCGAC |
| *HYP1* | GCGTCCCAAAGCACAAGCC | CATTGCTTGTAGAGCGGTTATCC |
| *HYP2* | GACGCCGTATTCGACGCAGG | AACTCCAGGGAACCGACTTTACC |
| *HYP3* | GCTGGCAACCCCTTGGCTGTAG | TGCCAGCGTCGTGAAGATG |
| *HYP5* | TTAGATGCGAATGAGAATGCGTATG | GGAGTTGTTTGAGTTCGATTTGATG |
| *HYP6* | CTCCACAGTCAGCAACAGTAACATTT | GGATGCGGCTGCTGTTACCT |
| *HYP7* | CGTGGGCTTCTTGGCCGCTAC | GGGTATCCGGTTGAGTTGGCTG |
| *HYP8* | CGGAGAGGAATGATCGCCC | GAGCCAGTAAGGAGAGACCATGTT |
| *HYP10* | TGCTGTGAAGGTGCAGACGAG | GATAGGCAATGTACAACTCCAGCAC |
| *HYP15* | GACAGAAGACAGCTAGATTGGGCTC | GATATTTTCCTGTGGTGAAATTGGTC |
| *HYP16* | CTGGGTGAAGCCACGGTTATCG | CTTGGTGGGCAATCTTGGTCG |
| *HYP17* | GTACAAGAAGTGCTTTCCGAACATCC | CAACGATCAGCTGCGACCAGGC |
| *HYP18* | GAACAAAGCCATCATCGGCATCC | GAGACCCTTCTTTTGGTCATCGG |
| *KIN2* | CGATGGGAACCTGAGGGTGTAG | CGCGGTGAGAAGGGTCTGCTTTG |
| *MFS5* | CAAGGAATGGGTGGCTGGAG | GGCGATGGCGTAGGTGACTGT |
| *OXR1* | CAGATGGGCGGACTATTAAGGCAG | CCGACGTAGTGTCCGTGGTGC |
| *PEP4* | TCCAGTAGACAACTTCCTCAACGCT | ATGAAGGTAGCAGGCGATGGAG |
| *PHO* | TATTGGAATTGTTACCACGGCTC | GGCCTAGGTACGCCCTTTCAG |
| *RPS1B* | GGACGGATCTTTGAGGTTTCG | AGTTTGCCATTTGCGGACCA |
| *SID4* | CGGCGGCAGGACACTCTATC | GCGGCGGACAGCAGAGTATG |
| *SNF3* | GAAATCTCCGTCGCGTAATCCTG | GCATGGCCATTGCTCCGTAG |
| *SOD3* | CACCTACCTTACCGATACCCAAT | GCAGTGTAAGTTACGTTAGGTGGG |
| *TDH1* | GTGGTCGTTGAGGGCGATGC | GACCATCAAGAAAGCCTCTGAGG |
| *TEF1* | GCTCTGCTTGCTTTCACCCTTG | TCTCCTTGTTCCAGCCCTTGT |
| *TEF3* | TGAAGAAGCAACTCGCAAACAA | GACAAGGGCAACAGCGGCAGC |
| *TRL1* | CAACATTGGTGCTGTCGTCGTTA | CAAACACAACTACTCCCCGATC |
| *YPS3* | ATGCTGAACATCAAATCGATCTC | TGCCTCGCAGTGTTTATAAAGCG |
| *ZRT1* | TTTCCATTGCCGTTGGCCTG | GAAGTCCGAACGCAGCCAGC |
